# Assessing the effectiveness and cost-effectiveness of audit and feedback on physician’s prescribing indicators: study protocol of a randomized controlled trial with economic evaluation

**DOI:** 10.1186/2008-2231-20-88

**Published:** 2012-12-06

**Authors:** Fatemeh Soleymani, Arash Rashidian, Rassoul Dinarvand, Abbas Kebriaeezade, Mostafa Hosseini, Mohammad Abdollahi

**Affiliations:** 1 Department of Pharmacoeconomics and Pharmaceutical Management, Faculty of Pharmacy, Tehran University of Medical Sciences (TUMS), Tehran, Iran; 2 National Committee on Rational Drug Use, Food and Drug Organization, Ministry of Health and Medical Education, Tehran, Iran; 3 Department of Health Management and Economic, School of Public Health, TUMS, Tehran, Iran; 4 Knowledge Utilization Research Center, TUMS, Tehran, Iran; 5 Department of Pharmaceutics, Faculty of Pharmacy and Nanotechnology Research Center, TUMS, Tehran, Iran; 6 Department of Toxicology and Pharmacology, Faculty of Pharmacy and Pharmaceutical Sciences Research Center, TUMS, Tehran, Iran; 7 Department of Epidemiology and Biostatistics, School of Public Health, TUMS, Tehran, Iran; 8 National Institute of Health Research, Tehran University of Medical Sciences, No 78, Italia Ave, Tehran, Iran

**Keywords:** Intervention study, Rational drug use, Audit and feedback, Printed educational material, Iran

## Abstract

**Background:**

Physician prescribing is the most frequent medical intervention with a highest impact on healthcare costs and outcomes. Therefore improving and promoting rational drug use is a great interest. We aimed to assess the effectiveness and cost-effectiveness of two forms of conducting prescribing audit and feedback interventions and a printed educational material intervention in improving physician prescribing.

**Method/design:**

A four-arm randomized trial with economic evaluation will be conducted in Tehran. Three interventions (routine feedback, revised feedback, and printed educational material) and a no intervention control arm will be compared. Physicians working in outpatient practices are randomly allocated to one of the four arms using stratified randomized sampling. The interventions are developed based on a review of literature, physician interviews, current experiences in Iran and with theoretical insights from the Theory of Planned Behavior. Effects of the interventions on improving antibiotics and corticosteroids prescribing will be assessed in regression analyses. Cost data will be assessed from a health care provider’s perspective and incremental cost-effectiveness ratios will be calculated.

**Discussion:**

This study will determine the effectiveness and cost-effectiveness of three interventions and allow us to determine the most effective interventions in improving prescribing pattern. If the interventions are cost-effective, they will likely be applied nationwide.

**Trial registration:**

Iranian Registry of Clinical Trials Registration Number: IRCT201106086740N1Pharmaceutical Sciences Research Center of TUMS Ethics Committee Registration Number: 90-02-27-07

## Background

Over the past decades, new generations of antibiotics, analgesics, corticosteroids and other medicines have been introduced to the pharmaceuticals market. At the same time, health care systems are confronted with a continuing pressure to provide high-quality care in the face of increasing costs and limited financial resources.

Due to the rising pharmaceutical expenditures in the world, governments are struggling to keep costs under control [1-3]. There are valid concerns about the rationality of use of medicines, mostly as a result of inappropriate physician prescribing. Physicians’ prescribing is the most common medical intervention with a high impact on health care costs [4]. Improving and promoting rational use of medicines contributes to a more efficient use of meager health care resources, as well as ensuring better population health outcomes.

Changing prescriber behavior is difficult and may require complex multilayered interventions [5]. Such interventions may include using audit and feedbacks, dissemination of printed educational materials, educational outreach visits, reminders, and opinion leaders among others [6,7]. Implementing the multilayered intervention is more costly and resource intensive. Hence, it is important to identify easy to implement an effective intervention to improve physicians’ prescribing. Several studies have been conducted to develop interventions and strategies to improve prescribing [8-13] but most of them have been carried out in high-income countries, sometimes with conflicting results [14]. Only limited high quality studies exist from low and middle-income countries.

According to a Cochrane Review, audit and feedback is defined as a summary of health care performance over a specified period of time with or without recommendations for a clinical action [15]. Evidence from 118 randomized controlled trials indicated that audit and feedback resulted in a modest improvement in care [15]. Grimshaw and colleagues [16] undertook a comprehensive review of the effects of using different strategies for implementing guidelines. They found that audit and feedback alone or combined with educational meetings and materials result in a modest improvement in the implementation of guidelines in comparison to no intervention group. Furthermore the studies on assessing the effectiveness of printed educational materials indicated only a small impact on practice, but there was no reliable assessment of the cost effectiveness of such programs. As the small benefits from dissemination of printed educational materials are achieved at relatively low costs, they may still be worthwhile interventions [17,18].

In Iran for many years, the problems of irrational drug use have been investigated by academic members of several universities [19-24]. In 1996, the National Committee of Rational Use of Drug (NCRUD) was established and a formal process of assessing physician prescribing started in the country. To make this possible, different data sources were developed that included a central data warehouse as well as access to insurance organization prescription datasets. Also different interventions including audit and feedbacks, dissemination of printed educational materials, public education and workshops and conferences were employed. Although these national plans were not subjected to formal evaluations, descriptive assessments suggest that they may have resulted in improving prescribing behavior, including a descending trend in the mean medicine items per prescription as an important indicator for rational drug use [25,26]. So far, no systematic and scientific evaluation has been conducted to determine the effectiveness and cost effectiveness of audit and feedback interventions in Iran. This trial is designed to test three different interventions for improving rational drug use indicators of general physicians, pediatricians and infectious disease specialists.

The main objectives of the study are:

1) Does the routinely conducted prescribing audit and feedback intervention influence general physicians, pediatricians and infectious disease specialists prescribing behavior in comparison to printed educational materials and no intervention?

2) Does a revised audit and feedback intervention improve prescribing behavior compared to the routinely conducted prescribing audit and feedback, printed educational materials and no intervention? At the same time, we evaluated cost-effectiveness of the interventions compared to the control groups.

## Methods

### Study design

**Figure 1:**
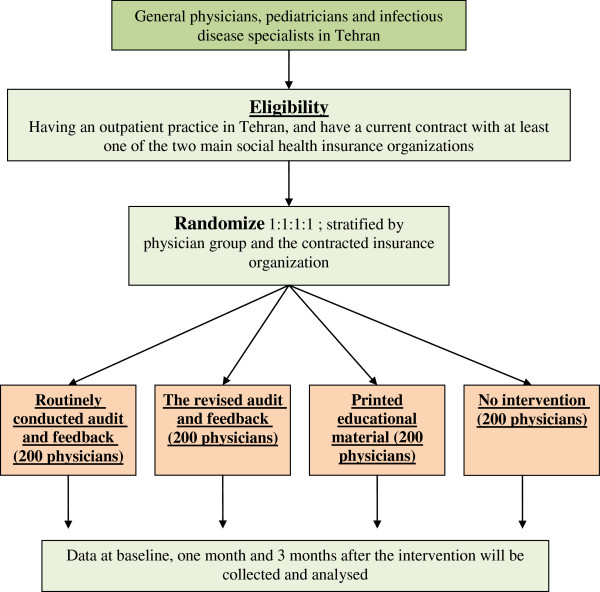
Trial flow proposed physicians enrollment and randomization procedures.

The study design is a four- armed randomized controlled trial with economic evaluation. It includes three different intervention arms and one control arm: routinely conducted audit and feedback, revised audit and feedback, printed educational materials (pamphlets), and a no intervention control group (see Trial Flow in Figure 1).

### Population and setting

The target population included general physicians, pediatricians and infectious disease specialists who have an outpatient practice in Tehran, and have a current contract with at least one of the two main social health insurance organizations.

### Details of the interventions

Routinely conducted audit and feedback involves a three-page summary of prescriptions indicators based on the analysis of insured prescriptions. The summary reports the prescribing indicators for each physician in comparison with their peer groups in the same area in a given time period (annual and quarterly). The first page is cover letter signed by the Secretary of the RUD committee in the region, consisting of general points about the common problems especially in the area which they practice. The second page includes physician’s profile such as name, specialty, medical council number, office address, the evaluation period and the insurance organization that have provided prescription data. Then, the prescription amount of main indicators will be listed in comparative tables that include the following:

1. Average medicine items per prescription

2. Average price per prescription (IR Rials)

3. Proportion of prescriptions including one medicine item (%)

4. Proportion of prescriptions including four or more medicine items (%)

5. Maximum medicine items in a prescription

6. Proportion of injectable medicine items per all prescribed items (%)

7. Proportion of prescriptions that includes at least one injectable medicine item (%)

Consequently, the prescription indicators for important drug groups such as antibiotics, corticosteroids and NSAIDs will be reported. These groups will be chosen based upon common prescribing problems in any given area. Moreover, the list of 10 most-prescribed drugs and 10 first most expensive drugs are reported in separate tables. Finally, 10 important drug interactions with high severity and intensity in the physician’s prescriptions are reported [27].

The revised audit and feedback has been developed for this trial based on current drug use problems in Iran. We developed our intervention based on four inputs: the current audit and feedback, a literature review, interviews with physicians, and the theoretical justifications acquired from the Theory of Planned Behavior (TPB).

We conducted a literature review to obtain feedback’ samples from other countries [28] and identifying current and important drug prescribing problems in our country [25,26]. Face-to-face interviews were conducted with16 physicians.

**Figure 2:**
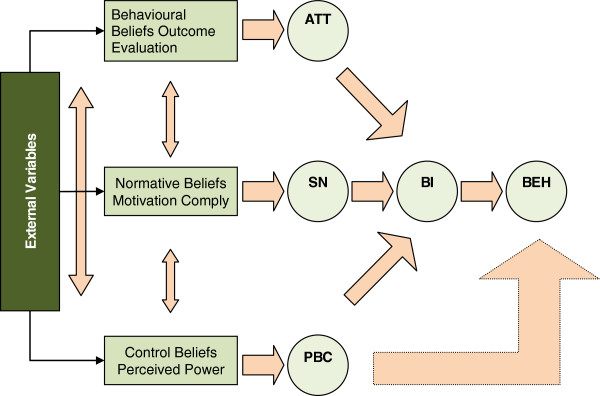
**Theory of Planned Behavior [**[6]**].**

The TPB [29] is a social cognition theory to provide a theoretical basis for explaining volitional behavior. The theory has important merits in explaining and understanding physician behavior, including prescribing [30-33]. The TPB suggests that individuals’ intention to perform behaviors is determined by their attitudes, perceived social or peer pressures (subjective norms) and perceived behavioral control [Figure 2.

The revised feedback form consisted of three pages. The form uses traffic light colors for reporting the prescribing indicators by physicians. Based on the findings from interviews, the revised feedback form was designed to look less crowded than the routinely conducted feedback and to ensure physicians’ attention was attracted towards important messages in the feedback. The indicators were also selected more objectively. The first page expresses the purpose of providing feedback and interpreting the colors used in the report and the importance of improving the situation of irrational prescribing signed by secretary of the committee. If dexamethasone and ceftriaxone injectable forms and an oral form of cefixime are at the beginning of the list of 10 most prescribed drugs, notice will be given. The revised feedback includes the following indicators on the first page:

1. Average medicine items per prescription

2. Average price per prescription (IR Rials)

3. Proportion of prescriptions including four or more medicine items (%)

4. Proportion of prescriptions that includes at least one injectable medicine item (%)

5. Proportion of prescriptions that includes at least one antibiotic medicine item (%)

6. Proportion of prescriptions that includes at least one corticosteroid medicine item (%)

In the second page, table of the most prescribed antibiotics in the country as prescribed by each physician are reported. Then, a graph reports all antibiotics prescribed by a physician and his/her peer group according to differentiate seasons. At the bottom of the second page, two important practice points related to prescribing antibiotics are presented.

Table of prescription amount of most prescribed systemic corticosteroids in the country as prescribed by the physician are presented on the third page. Then, a graph reports all injectable forms of medicines prescribed by the physician and compares it with his/her peer group. At the bottom of the third page, two important practice points related to prescription of injectable forms of medicines and corticosteroids are presented.

### Printed educational material (pamphlet)

The educational material was also developed using literature review, interviews with physicians, with theoretical attentions based on the TPB. Most physicians participating in the interviews emphasized on the usefulness of a concise pamphlet with a practical training content. Hence, a 4-page A5 size pamphlet was designed. The pamphlet provides practical tips on prescribing antibiotics, corticosteroids and injectable dosage forms and the current status of utilization of these medicines in the country. It also documents examples of serious adverse effects reported from inappropriate usage of ceftriaxone injections and serious complications after dexamethasone injections.

### Study setting and participants

The study is conducted in Tehran. All general physicians, pediatricians and infectious disease specialists who have an outpatient practice in Tehran, issue a minimum of 100 prescriptions per month, and have a current contract with at least one of the two main social health insurance organizations are eligible for inclusion in the study.

### Sample size calculation and allocation to the intervention arms

Considering that about 14% of outpatient prescriptions in Tehran contain dexamethasone injections and expecting that the intervention may reduce such prescriptions by 10%, we aimed to recruit 200 participants in each study arm (i.e. a total 800 participants).

As the interventions will be delivered to all the physicians allocated to the study arms (effectiveness trial) and that we used routinely collected data to assess the effects, the prospect of loss to follow was low.

To allocate the participants to the study arms, we first prepare a list of all eligible physicians working in Tehran (about 6000 physicians). The physicians will be grouped into the three physician groups (i.e. general physicians, pediatricians, infectious disease specialists). Within each group we will randomly select the required number of physicians, proportional to the size of each group. Using a random number generator, the participants will be allocated to the study arms, and use the physician group and the contracted insurance organization as the stratifying factors. As a result we will have, in each study arm, 175 general physicians, 24 pediatricians, and four infectious disease specialists (totaling 203 physicians in each arm). Random numbers will be generated by a member of the team who is not involved in the delivery of the interventions.

### Outcome measures

The main outcome measures for assessing the effectiveness of the interventions, the percentage of change in the proportion of dexamethasone injectable form, oral form of cefixime and amoxicillin which prescribed by general physicians, pediatricians and infectious disease specialists will be considered.

Costs are assessed from a health care provider perspective. We include all identifiable costs related to the implementation of the interventions. Our analysis therefore focuses on the incremental costs of developing and implementing audit and feedback and printed educational material interventions. Recurring and non-recurring costs are considered. Nonrecurring costs include revising the computer software and designing the new format of the feedback, and the pamphlet. Recurring costs include printing materials, travel costs, postage costs and other administrative tasks.

### Study procedures and data collection

Data at baseline, one month and 3 months after the intervention will be collected.

### Statistical and cost-effectiveness analyses

Demographic and baseline analyses will be conducted to ensure random allocation procedure has been effective in producing comparable study arms. Chi-square, *t*-test, repeated measure ANOVA and logistic regression analyses will be the primary modes of analyzing the results. As the primary outcomes, we will assess the effects of the study arms on the proportion of prescriptions containing each of the target prescribed medicines. We will also assess the effects of the interventions on the mean cost per prescription, and the mean number of items prescribed per prescription. Appropriate interaction analyses will be conducted to assess the interactions between the selected variables and the interventions. A fixed-effect two-level analysis approach will be followed in which prescriptions will be used as the first level and the physicians as the second level of data.

Cost data will be assessed using the actual costs incurred in the study, from a health care provider view point. Incremental cost-effectiveness ratios will be calculated and sensitivity analyses will be also conducted to assess the effects of changes in important variables in the cost-effectiveness of the interventions.

Intention-to-treat analyses will be carried out with physicians as the primary analysis approach. We will also conduct a per protocol analysis as a sensitivity analysis.

### Ethical considerations

These data have been extracted from the Health Insurance Organization claims database and are provided confidentially for each physician for personal review. This study was approved by the Ethics Committee of Pharmaceutical Sciences Research Center of TUMS and has been registered with the Center for International Registration of Clinical Trial.

## Discussion

A thorough evaluation of the intervention for improving and promoting rational drug use may bring important benefits, whereas inappropriate prescribing may lead to undesirable consequences. We see this trial as a necessary prerequisite for the development and implementation of a cost-effective intervention that will improve and promote rational drug use in Iran.

This study will determine the effectiveness of the three interventions, and allow us to determine what kind of intervention will be the most effective in improving prescribing pattern. This work will be the first to assess the effectiveness and cost-effectiveness of the routinely conducted audit and feedback that has been widely used in the country.

The risk of contamination between the study arms is a potential limitation of the study. However as the study sample is selected from a large number of physicians working in Tehran, the risk of contamination should be small. Additional potential limitation of the study may arise due to concurrent interventions that might happen by the responsible authorities within the Ministry of Health and Medical Education, as well as the universities in charge of health care in Tehran. We will follow such activities throughout the study, and will assess their potential impacts on the study findings, if they occur.

We believe that the current proposal may have other benefits. We expect that it will lead to the development of focused interventions that could greatly promote rational drug use. Changing physician’s behavior is challenging and involves attention to the views and concerns of practicing physicians. We attended to the physicians’ views while developing our interventions. In addition, this trial shall establish whether the design of targeted feedback can be used as a novel format of interventions for promoting rational drug use. We hope that, if our interventions are cost-effective, they shall be widely applied nationwide. The interventions may also be of use in middle income countries with appropriate infrastructures.

## Competing interests

Authors have no conflict of interest. Since MA is Editor-in-Chief of DARU, all the review process is handled by one of Section Editors.

## Authors' contributions

AR, FS designed the study with contributions from RD, AK, MH, and MA. FS collected data, registered the study and wrote the first draft of the manuscript. FS and AR revised the manuscript based on comments from the other authors. All the authors approved the final manuscript.
